# Detect tissue heterogeneity in gene expression data with BioQC

**DOI:** 10.1186/s12864-017-3661-2

**Published:** 2017-04-04

**Authors:** Jitao David Zhang, Klas Hatje, Gregor Sturm, Clemens Broger, Martin Ebeling, Martine Burtin, Fabiola Terzi, Silvia Ines Pomposiello, Laura Badi

**Affiliations:** 1 Roche Pharma Research and Early Development, Pharmaceutical Sciences, Roche Innovation Center Basel, F. Hoffmann-La Roche Ltd, Grenzacherstrasse 124, Basel, 4070 Switzerland; 2 Inserm U1151, Université Paris Descartes, Institut Necker Enfants Malades, Hôpital Necker Enfants Malades, 149, Rue de Sèvres, Paris, 75015 France; 3 Present address: Peter-Rot-Strasse 84, Basel, 4058 Switzerland

**Keywords:** Gene expression, Quality control, Wilcoxon-Mann-Whitney test, Gene-set enrichment analysis

## Abstract

**Background:**

Gene expression data can be compromised by cells originating from other tissues than the target tissue of profiling. Failures in detecting such *tissue heterogeneity* have profound implications on data interpretation and reproducibility. A computational tool explicitly addressing the issue is warranted.

**Results:**

We introduce *BioQC*, a R/Bioconductor software package to detect tissue heterogeneity in gene expression data. To this end *BioQC* implements a computationally efficient *Wilcoxon-Mann-Whitney* test and provides more than 150 signatures of tissue-enriched genes derived from large-scale transcriptomics studies.

Simulation experiments show that *BioQC* is both fast and sensitive in detecting tissue heterogeneity. In a case study with whole-organ profiling data, *BioQC* predicted contamination events that are confirmed by quantitative RT-PCR. Applied to transcriptomics data of the Genotype-Tissue Expression (GTEx) project, *BioQC* reveals clustering of samples and suggests that some samples likely suffer from tissue heterogeneity.

**Conclusions:**

Our experience with gene expression data indicates a prevalence of tissue heterogeneity that often goes unnoticed. *BioQC* addresses the issue by integrating prior knowledge with a scalable algorithm. We propose *BioQC* as a first-line tool to ensure quality and reproducibility of gene expression data.

**Electronic supplementary material:**

The online version of this article (doi:10.1186/s12864-017-3661-2) contains supplementary material, which is available to authorized users.

## Background

Gene expression data has become indispensable in modern drug discovery. It reveals biological processes underlying pathogenesis and sheds light on mode-of-action and potential safety liabilities of drug candidates. However, its value in catalyzing new medicines is shadowed by limited reproducibility [1]. From the (re-)analysis of a large number of internal and external studies we observe that tissue heterogeneity, defined as unintended profiling of cells of other origins than the target tissue of profiling, is a common source of variance that exacerbates the irreproducibility.

Many factors can cause tissue heterogeneity in gene expression profiling experiments. Some are attributed to underlying physiological or pathological processes, for example infiltration of immune cells into solid organs or tumors. Some are associated with the challenge of dissecting adjacent tissues such as coronary artery and cardiac muscle. In other cases heterogeneity is due to human errors such as contamination or mislabeling. Independent of root causes, tissue heterogeneity in gene expression data should be identified as early as possible to prevent it impacting downstream analysis. No software tool, however, fulfills this purpose to our best knowledge.

Various computational approaches may address the issue and they can be broadly classified into *unsupervised* and *supervised* algorithms. Unsupervised algorithms, such as principal component analysis (PCA) and its variants, may detect tissue heterogeneity without prior knowledge [2]. Nonetheless unsupervised algorithms are only sub-optimal in this setting because (a) they cannot reveal likely sources of heterogeneity, (b) they cannot work with single sample, and (c) their application is limited to cases where few samples are heterogeneous, since otherwise it becomes difficult to distinguish heterogeneous from homogeneous samples.

Supervised algorithms overcome these restrictions by incorporating prior information such as sets of preferentially expressed genes [3] or reference profiles of purified cells [4]. While several supervised methods are available to quantitatively estimate the composition of cell types (reviewed in [5]), their application in first-line quality control is limited because most tools rely on a substantial amount of prior information which is often not available, for instance profiles of each single cell type that may present. In addition, these tools often use complex models such as expectation-maximization or quadratic programming which demand significant computational resources and therefore are hardly scalable to large-scale datasets, for instance the Genotype-Tissue Expression (GTEx) project [6, 7] which contains more than 8500 profiles in its current (6^*th*^) version.

To allow efficient and sensitive detection of tissue heterogeneity, we introduce the software package *BioQC*. It takes gene expression data as input, performs statistical tests with tissue-enriched gene signatures that come with the package, and reports enrichment scores of more than 150 tissue signatures for each sample. High scores of tissues other than the target tissue of profiling indicate heterogeneity and consequently possible infiltration or contamination events.

## Implementation

*BioQC* follows the supervised approach: it provides sets of genes that are preferentially expressed in one or few tissues (*tissue-enriched genes* hereafter), and an efficient algorithm to test enrichment of tissue-enriched genes in expression data.

### Tissue-enriched genes

We derived 155 sets of tissue-enriched genes (*tissue signatures* hereafter) from four datasets: the *Neurocrine Biosciences (NB) CNS dataset* [8], the *GNF Gene Expression Atlas* [9], both based on the *Affymetrix* microarray technology, and sequencing-based *RNASeq Atlas* [10] and Illumina BodyMap 2.0 (GEO Accession Number GSE30611 [11]). For preprocessing we normalised microarray signals with the *MAS5* method [12] and converted sequencing read counts into the unit of *copies per million reads* [13]. Expression signals are averaged in case more than one samples are available for each tissue. Both sequencing-based datasets are merged by removing batch effects using a linear model in order to achieve a wide coverage of tissues comparable with microarray-based datasets.

*Gini index* [14] was used to identify tissue-enriched genes. Given an *m*×*n* expression matrix with *m* genes and samples of *n* tissues, Gini index for gene *i* is defined as

1 $$ G_{i}=\frac{1}{n}\left(n+1-2\left(\frac{{\sum\nolimits}_{j=1}^{n}(n+1-i)x'_{ij}}{{\sum\nolimits}_{j=1}^{n}x'_{ij}}\right)\right),  $$

where $x^{\prime }_{ij}$ is the *j*th value in the non-descending ordered vector of *x*_*i*·_ (*i*=1,…,*m*,*j*=1,…,*n*). Gini index ranges between 0 and 1, depending on whether the gene is ubiquitously and uniformly present or absent in all tissues (*G*=0) or is exclusively expressed in one tissue (*G*=1) or in between (0<*G*<1).

We consider gene *i* enriched in tissue *j* if $G_{i} \geqslant 0.7$ and *x*_*ij*_ ranks among the top three in *x*_*i*·_. Signatures of identical tissues derived from both microarray datasets are merged, while microarray-based and sequencing-based signatures are kept separate so that users can benefit from the high sensitivity offered by the sequencing technology. Users can define their own signatures using other datasets with functionalities implemented in *BioQC* or with other statistical methods.

### Validation of tissue-enriched genes

We took a three-tier approach to validate the tissue signatures. First, in order to assess their robustness against batch effects, we applied *surrogate variable analysis* [15] to both *GNF* and *NB* datasets to detect uncaptured batch effects (not applicable to sequencing-based datasets where only one sample is available for each tissue). Based on batch-effect-corrected data, we generated a new set of signatures using the procedure described above. We applied the *BioQC* algorithm using both signature sets to the GTEx gene expression database [7] and found that the results are highly similar (Additional file 1: Figure S1), which suggests that the *BioQC* tissue signatures are robust with regard to potential batch effects in the source data.

Next, we used biological knowledge to test the validity of the tissue signatures using *Roche Controlled Vocabulary* (*RCV* hereafter), a recently developed, simplified ontology system of annotating human genes with biological processes defined by the Gene Ontology consortium [16]. We applied the *BioQC* algorithm, using both tissue-enriched gene sets and RCV gene sets, to the GTEx dataset, and found that for many tissues that are included in GTEx, expected tissue signatures are co-enriched with relevant biological processes. For instance, most tissue signatures associated with the nervous system are co-clustered with genes associated with neuronal biological processes (Additional file 1: Figure S2). The observation suggests that the tissue signatures are biologically relevant.

Last but not least, to test the validity of using Gini index to identify tissue signatures, we applied an independent algorithm, *limma* [17], to the GTEx dataset (*limma* was prefered to negative-binomial-model based methods such as *DESeq2* and *edgeR* due to computational efficiency and the compatibility of results). We identified signatures of 29 tissues that are present in GTEx using very stringent filters. For those tissues that are present in the BioQC tissue signatures, we observed moderate to strong overlapping and comparable peformance with *BioQC* on a large collection of gene expression data (manuscript in preparation). It suggests that *BioQC* tissue signatures are consistent with signatures generated from an independent dataset by an alternative statistical method.

### The algorithm of *BioQC*

*BioQC* implements a computationally efficient *Wilcoxon-Mann-Whitney* test (*Wilcoxon* test hereafter) [18]. The algorithm is accelerated by (a) an approximate test procedure, (b) implementation of the core algorithm in *C* programming language, and (c) elimination of futile sorting operations. Improvement that we made over standard implementations is detailed in Additional file 2: Document 1.

Given a gene expression profile and a tissue signature, *BioQC* tests by default whether expression of genes in the signature ranks higher than expression of genes not in the signature. Users can test negative enrichment or two-sided (either positive or negative) enrichment, too.

*BioQC* reports an enrichment score of each tissue signature for each sample in the form of |*l* *o* *g*_10_ *p*| (absolute log10-transformed *p*-value of *Wilcoxon* test). Given expression profiles of *n* samples as input and *s* tissue signatures, *BioQC* outputs an *s*×*n* matrix with scores ranging between 0 (no enrichment) and theoretically positive infinity (strong enrichment). By examining *BioQC* results and comparing them with target tissues of profiling users can inspect heterogeneity and generate hypotheses about the causes.

Although this work focuses on its application to detect tissue heterogeneity, we note that *BioQC* can be used as a generic gene-set enrichment analysis tool (manuscript in preparation).

## Results

We apply *BioQC* to simulated and real-world datasets to demonstrate its use. All computations are performed on a single thread of a 4-core laptop with 8G memory running R-3.2.0 in 64-bit Linux MINT (version 16) if not otherwise specified.

### Simulation studies

We performed three simulation experiments to study the efficiency and sensitivity of *BioQC*.

#### Simulations with model-generated data

The first simulation study probes the speed of *BioQC*. We propose a simple model to generate random gene expression profiles of approximately 22,000 genes with each gene following the normal distribution $\mathcal {N}(0,1)$. We let the model generate five datasets with varying sample sizes (*n*=1,5,10,50,100), applied *BioQC* and timed the runs. For benchmarking we applied the native implementation of *Wilcoxon* test in *R* (function *wilcox.test*) to the same dataset.

**Fig. 1:**
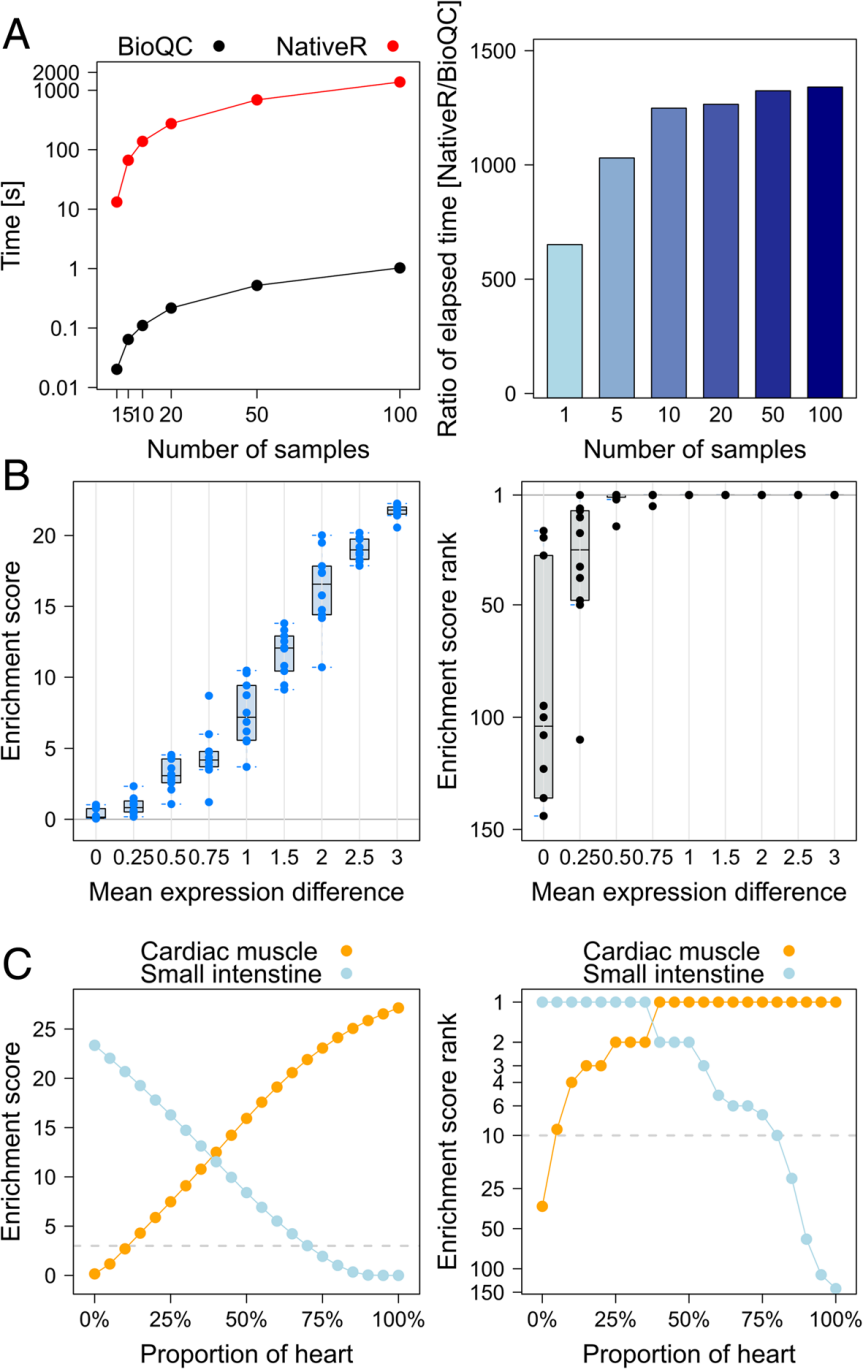
Results of simulation studies **a** Speed benchmark. *Left panel*: running time of *BioQC* and R-native *Wilcoxon* test with simulated datsets of increasing sample sizes. *Right panel*: ratio of running time between the two implementations. **b** Sensitivity of *BioQC* revealed by simulations with model-generated data. *Left panel*: whisker-box-plot of *BioQC* enrichment scores of the selected gene set (*Y-axis*) against the average expression differences of genes in the set compared with genes not in the set (*X-axis*). *Right panel*: whisker-box-plot of ranks of enrichment scores. **c** Sensitivity of *BioQC* revealed by simulations with real-world data. *Left panel*: Enrichment scores of cardiac-muscle- and small-intestine-enriched genes as canine heart and jejunum samples are mixed with varying weights. *Right panel*: Ranks of enrichment scores plotted against varying weights

Both implementations delivered identical numeric results and their memory use is comparable. However, while it takes the native implementation 20 minutes to analyze 100 profiles, it takes *BioQC* just about one second (Fig. 1 a left panel). Quantitatively *BioQC* accelerates the *Wilcoxon* test by a factor between 500 and 1000 for gene expression data (Fig. 1 a right panel). The results suggest that *BioQC* is computationally efficient and applicable for large-scale datasets even on laptop computers.

Next, we investigated the sensitivity of *BioQC* towards expression changes of tissue-enriched genes. To this end, we adapted the simple model used above: while keeping random-number generators of other genes unchanged, expression levels of genes in a randomly chosen signature (ovary with 43 genes) were drawn from a series of distributions $\mathcal {N}(\mu,1)$ with *μ* varying from 0 to 3. We generated ten samples for each distribution and applied *BioQC*.

We observed that the average enrichment score of ovary increases steadily as *μ* increases (Fig. 1 b left panel), and as soon as *μ* reaches or exceeds 1, the ovary signature ranks first among all signatures in all simulated samples (Fig. 1 b right panel). Repeated experiments with other tissue signatures produced consistent and comparable results (data not shown). In other words it is sufficient for a tissue signature to rank first if average expression of its genes increases by one standard deviation. It suggests that *BioQC* is sensitive to even mild changes in expression of tissue-enriched genes.

We note nevertheless that the sensitivity test above suffers from the limitations that (a) the distributions of gene expression are not physiological and (b) elevated expression of tissue-enriched genes is not quantitatively associated with severity of contamination. To corroborate our findings we next performed simulations with real-world data.

#### Simulation with real-world data

We simulated tissue heterogeneity *in silico* with a transcriptomics study in *Canis lupus familiaris* (domestic dog) by Briggs *et al.* [19]. In this study the authors constructed a compendium of canine normal tissue gene expression, which includes 39 samples of 10 tissues from four dogs (four samples of liver, kidney, heart, lung, cerebrum, lymph node, spleen, jejunum, and skeletal muscle; three samples of pancreas).

Prior to simulation experiments we first applied *BioQC* to the dataset to test whether its results match information provided by the authors. In [19], the authors performed hierarchical clustering of both canine and human data and reported a ’remarkable similarity’ of normal tissue gene expression profiles between the two organisms. Indeed, *BioQC* confirmed the consistency in 36 (92%) cases in which canine tissues reported by the authors match the highest-ranking human tissues reported by *BioQC* (Table 1 in Additional file 3: Document 2). Among the three discrepant cases, one was originally labeled as prefrontal cortex but found by *BioQC* to resemble the spinal cord most (sample ID GSM502573), and the other two samples were labeled as lung but found most similar to monocytes (GSM502594 and GSM502596). Interestingly, the authors’ findings seem to be supportive of our observations: GSM502573 is an apparent outlier in the PCA analysis (Fig. 1 a in [19]), and the authors noted that average profiles of canine lung and spleen samples, unlike other tissues, do not cluster together with respective human tissues in hierarchical clustering and show similarity with each other (Fig. 2 a in [19]).

**Fig. 2:**
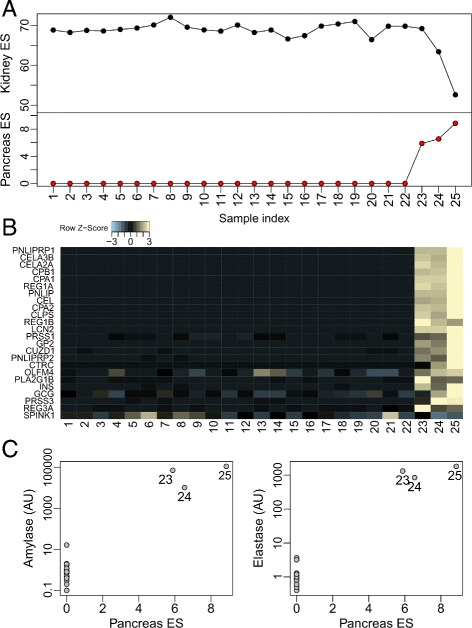
*BioQC* detects pancreas contamination of mouse kidney samples **a** Enrichment scores (ES) of kidney and pancreas signatures. **b** Normalised microarray signals of pancreas-enriched genes (zero mean and one standard deviation per gene). **c** Expression of amylase and elastase detected by qRT-PCR, with indices of contaminated samples labeled. AU: Arbitrary Unit

While it remains unknown what causes the observed heterogeneity, for the purpose of simulation the three samples were excluded. We then calculated an average expression profile for each tissue using the remaining 36 samples and simulated contamination by creating weighted linear combination of expression profiles of pairs of tissues, a procedure referred to as *mixing* hereafter.

Mathematically, given the expression profile of tissue A (**Y**_*A*_) and B (**Y**_*B*_), mixing generates a new profile **Y**=*ω* *Y*_*A*_+(1−*ω*)*Y*_*B*_ with *ω*∈ [0,1]. As *ω* approaches 1, the new profile mimics contamination of A by B; when *ω* approaches 0, it mimics contamination of B by A.

To illustrate the idea we applied *BioQC* to mixed profiles of heart and jejunum and visualize the results in Fig. 1 c. As the weight of heart increases, enrichment score of small intestine decreases while that of cardiac muscle increases. With as little as 10% contamination by heart in jejunum, enrichment score of cardiac muscle is above 3.0 (corresponding to *p*<10^−3^ of *Wilcoxon* test) and ranks 4^*th*^ out of 155 tissue signatures. With 25% contamination, enrichment score of cardiac muscle exceeds 7.0 and ranks second. These results suggest that *BioQC* is very sensitive towards heart contamination in jejunum.

Intriguingly, we observe an asymmetry in the sensitivity: it takes about 20-30% jejunum contamination in the heart sample to make the enrichment score exceed 3.0 or rank among the top ten. The asymmetry is likely caused by the relatively high expression of heart-enriched genes compared with small-intestine-specific genes in the respective tissue (Figure 4 in Additional file 3: Document 2). Despite of the asymmetry, we observe a strong leap of small-intestine signature’s rank as its proportion further increases. In practice when one of several heart samples is contaminated by jejunum, an aberrant higher rank of the jejunum signature can be a warning sign of tissue heterogeneity to the software user.

**Fig. 3:**
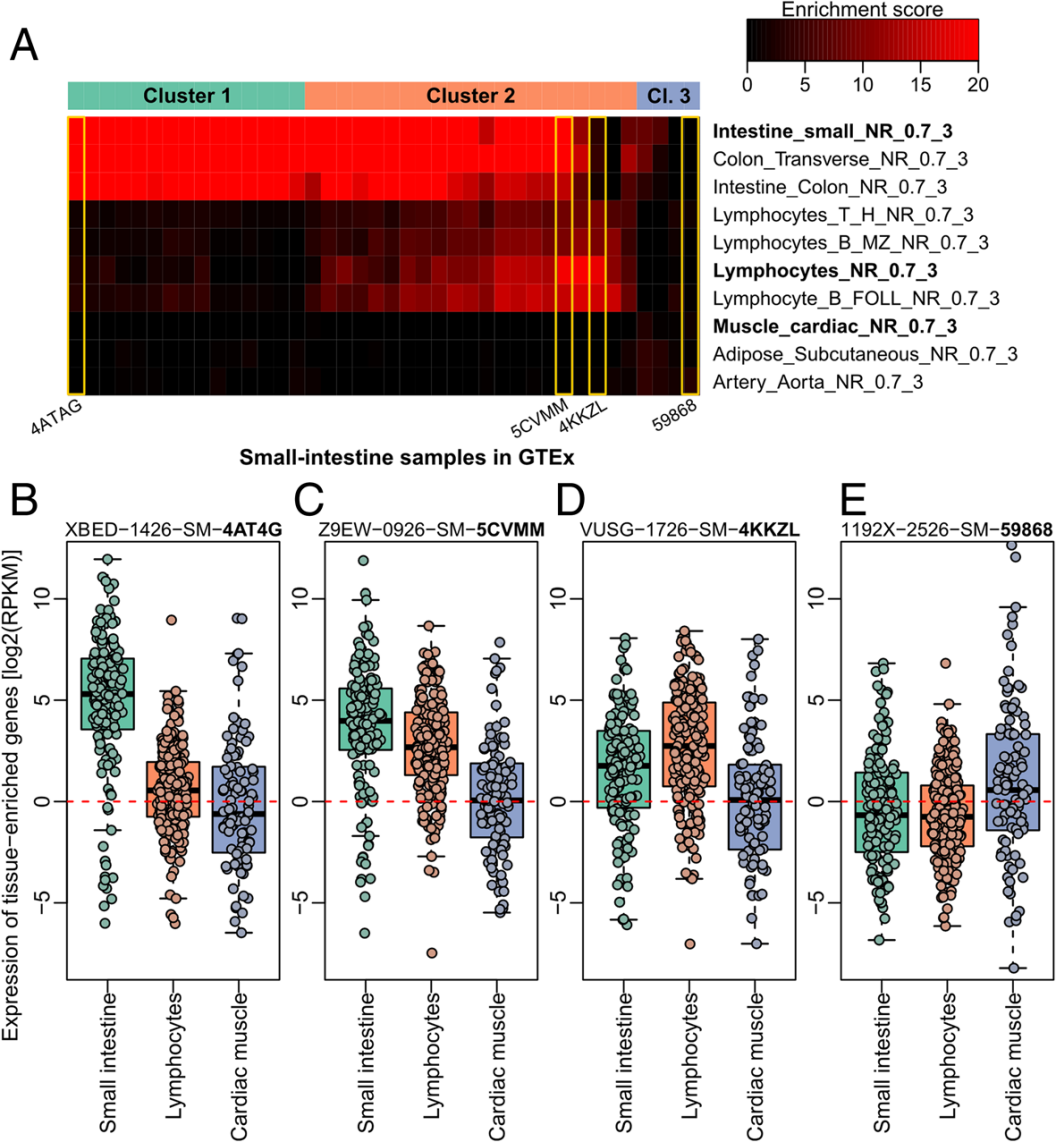
*BioQC* reveals sample clustering and tissue heterogeneity of small-intestine samples in GTEx **a** Tissue enrichment scores reported by *BioQC* when applied to small-intestine samples in GTEx. Samples are shown in columns and clustered by correlation-based hierarchical clustering. Ten tissue signatures with the highest average scores are shown in rows. Expression profiles of selected tissue signatures (*with bold row names*) in representative samples (*in yellow boxes*) are visualized below. The representative samples are labeled by the last five digits/letters of respective GTEx sample identifiers. **b**–**e** Whisker-box-plots of genes enriched in small intestine, lymphocytes, and cardiac muscle in representative samples. Each *dot* represents one signature gene. *Dash lines* indicate RPKM equal to one which represents an arbitrary threshold of low gene expression. RPKM: Reads Per Kilobase per Million mapped reads

Following this example, we mixed all pairs of canine tissues and found that on average *BioQC* is able to detect heterogeneity with 20% or more contamination (enrichment score $\geqslant 3.0$ or rank ≤10, Figure 3 in Additional file 3: Document 2). Tissues like liver and heart with highly expressed enriched genes can be more easily detected as sources of contamination with the proportion as low as 12%.

In summary, simulation studies with model-generated and real-world data demonstrate that BioQC is scalable and sensitive in detecting tissue heterogeneity. Since the simple statistical models that we used may not fully cover the complexity of tissue heterogeneity, we now turn to test *BioQC* with further real-world gene expression datasets.

### Case study with whole-organ profiling data

We applied BioQC to a dataset generated in a Roche research program. In this study twenty-five mouse whole-kidney samples were taken after diverse treatment regimen including operation and medication (details in Additional file 4: Document 3). Genome-wide gene expression profiling was performed with *Affymetrix* Mouse Genome 430 2.0 microarray.

Results of *BioQC* are visualized in Fig. 2 a. As expected, kidney-enriched genes ranked first among all tissue signatures in all samples. In three samples (index 23, 24 and 25), however, the pancreas signature scored much higher than in other samples. As we examined genes in the signature, we observed substantial expression of many genes including insulin (*INS*), glucagon (*GCG*), and pancreatic carboxypeptidase A1 (*CPA1*) in the three samples (Fig. 2 b). This made us suspect that these samples might be contaminated by traces of pancreas.

To validate the hypothesis, we quantified expression of amylase (*AMY1A*) and elastase (*CELA1*), both highly expressed in pancreas and absent in kidney according to GTEx [7] and Human Protein Atlas [20], with quantitative RT-PCR. We could indeed detect specific and substantial expression of both genes in the samples suspected of contamination (Fig. 2 c). Based on these results, we decided to exclude the three samples from downstream analysis. Exploratory analysis reveals that if the heterogeneity was overlooked and the contaminated samples were not removed, several pancreas-enriched genes would erroneously show strong differential expression in certain comparisons (Figure 4 in Additional file 3: Document 2).

In summary, the case study underlines the power of *BioQC* to detect tissue heterogeneity in gene expression data.

### *BioQC* applied to GTEx gene expression data

Finally we assessed tissue heterogeneity in a small subset of GTEx gene expression data by applying *BioQC* to small-intestine samples (*n*=40). *BioQC* revealed three clusters of samples based on enrichment of tissue signatures (Fig. 3 a). Cluster 1 is highly enriched of the small-intestine signature and almost devoid of enrichment of signatures of other tissues (Fig. 3 b). Samples in cluster 2 display decreased enrichment of the small-intestine signature and increased enrichment of the lymphocyte signature (Figs. 3 c and d). Surprisingly, samples in cluster 3 show enrichment of neither small-intestine nor lymphocyte signature, but increased expression of muscle-enriched genes (Fig. 3 e). It is noteworthy that exploratory analysis showed no significant association between the clustering and any of the clinical parameters reported by the GTEx consortium (age, sex, death causes, *etc.*; *χ* ^2^ test, significance threshold *α*=0.05).

Causes underlying the heterogeneity are obscure to us and may require substantial efforts to be clarified. However, one may speculate that an over-representation of intraepithelial lymphocytes or Peyer’s patch, due to either physiological or pathological courses, may contribute to the enrichment of the lymphocyte signature in samples of cluster 2. Enrichment of the muscle signature in cluster 3 may be caused by over-proportional mucosa or other cells of muscle’s origin. Despite the uncertainty of causes, gained information of sample heterogeneity can enhance the quality of downstream analysis. For example, results of *BioQC* can be injected into a weighted statistical model to identify genes that are preferentially present in small intestine, which may reveal specific cell-surface receptors that allow tissue-targeted drug delivery. Deprioritising samples outside of cluster 1 is likely to improve the specificity of identified targets.

In summary, *BioQC* reveals clustering of samples and tissue heterogeneity of different severity in a subset of GTEx data. Researchers using GTEx and similar resources are advised to perform quality control with *BioQC* before pursuing further analysis of data.

## Discussions and conclusions

While the impact of some factors underlying tissue heterogeneity may be minimized by taking greater care in experiment design, tissue dissection, and sample handling, factors such as immune cell infiltration are unlikely controllable. If overlooked, tissue heterogeneity may cause gene expression studies irreproducible because the heterogeneity is unlikely to be identical in an independent experiment.

Therefore we propose applying *BioQC* as a first-line quality control to detect tissue heterogeneity. *BioQC* tests enrichment of tissue-enriched genes sample-by-sample and suggests possible sources of heterogeneity, both of which are not possible for unsupervised methods like PCA. Compared with other supervised methods, *BioQC* comes with the required prior information and is extremely efficient. Extrapolating from the observation that *BioQC* needs about one second to analyze 100 genome-wide gene expression profiles, we estimate that it would take *BioQC* only about six hours to analyze the entire GEO database, which collects about 2.0 million gene expression profiles as of November 2016, with one CPU-thread (excluding the time of downloading, preprocessing, *etc.*). In light of its favorable scalability and sensitivity, we believe *BioQC* is suitable for small- and large-scale gene expression studies.

We expect tissue signatures to be applicable across closely related species as it was shown elsewhere that within mammalians the expression patterns of protein-coding genes are more conserved between species than between major organs [21, 22]. The human derived tissue signatures are already applied successfully to tissue expression data from multiple vertebrate model species including macaque, pig, dog, rodents, and zebrafish (data not shown). The simulation study using dog tissue expression data exemplifies such an analysis.

We have integrated *BioQC* in our gene expression analysis pipeline since three years to routinely detect tissue heterogeneity in internal and external studies. It has raised warning flags in many datasets independent of the target tissue of profiling, organism, experiment design, profiling platform and laboratory. While a few suspects were validated such as the ones in our case study, most findings were regrettably not followed up due to limited capacity and material unavailability. With more expression profiling data generated worldwide everyday, we wish that *BioQC* can help experimentalists and data analysts alike to improve the quality and reproducibility of gene expression studies.

## Availability and requirements

**Project name:** BioQC;

**Project home page:** http://accio.github.io/BioQC/;

**Operating systems:** Unix, Linux, Mac OS and Windows;

**Programming language:** GNU-R and C;

**Other requirements:** R and Bioconductor installation;

**Licence:** GPL-3.

## Additional files

Additional file 1 Supplementary Figures. (PDF 592 kb)

Additional file 2 Supplementary Document 1. This document can also be assessed on the *BioQC* website under [27] respectively. (ZIP 98 kb)

Additional file 3 Supplementary Document 2. This document can also be assessed on the *BioQC* website under [28] respectively. (ZIP 305 kb)

Additional file 4 Supplementary Document 3. This document can also be assessed on the *BioQC* website under [29] respectively. (ZIP 325 kb)

## Abbreviations

GEO: The gene expression omnibus database
GTEx: The genotype-tissue expression project
PCA: Principal component analysis
RT-PCR: Reverse transcription polymerase chain reaction
