# Correction to: Detect tissue heterogeneity in gene expression data with BioQC

**DOI:** 10.1186/s12864-018-4940-2

**Published:** 2018-07-30

**Authors:** Jitao David Zhang, Klas Hatje, Gregor Sturm, Clemens Broger, Martin Ebeling, Martine Burtin, Fabiola Terzi, Silvia Ines Pomposiello, Laura Badi

**Affiliations:** 1 Roche Pharma Research and Early Development, Pharmaceutical Sciences, Roche Innovation Center Basel, F. Hoffmann-La Roche Ltd, Grenzacherstrasse 124, 4070 Basel, Switzerland; 2 Institut Necker Enfants Malades, Inserm U1151, Université Paris Descartes, Paris Hôpital Necker Enfants Malades, 149, Rue de Sèvres, 75015 Paris, France; 3 Present Address: Peter-Rot-Strasse 84, 4058 Basel, Switzerland

## Correction

After the publication of this work [1], a mistake was noticed in the Eq. 1. Given an *m*  ×  *n* expression matrix with *m* genes and samples of *n* tissues, the correct definition of the *Gini index* for gene *i* is:

1 $$ {G}_i=\frac{1}{n}\left(n+1-2\left(\frac{\sum_{j=1}^n\left(n+1-j\right){x}_{ij}^{\prime }}{\sum_{j=1}^n{x}_{ij}^{\prime }}\right)\right), $$

where ${x}_{ij}^{\prime }$ is the *j*th value in the non-descending ordered vector of *x*_*i*⋅_(*i* = 1, …, *m*, *j* = 1, …, *n*). In the original version of the manuscript, the variable *j* in the parentheses of the nominator was erroneously written as *i*.

The authors apologize for the mistake and thank Mr. Tao Fang for pointing out this mistake.
