# Supplementary material for: Detect tissue heterogeneity in gene expression data with BioQC
Source: BMC Genomics. 2017 Apr 4;18:277. doi: 10.1186/s12864-017-3661-2 (PMC5379536; doi:10.1186/s12864-017-3661-2)
Supplement: Supplementary file 1 — Supplementary Figures. (PDF 592 kb) [file 12864_2017_3661_MOESM1_ESM.pdf]

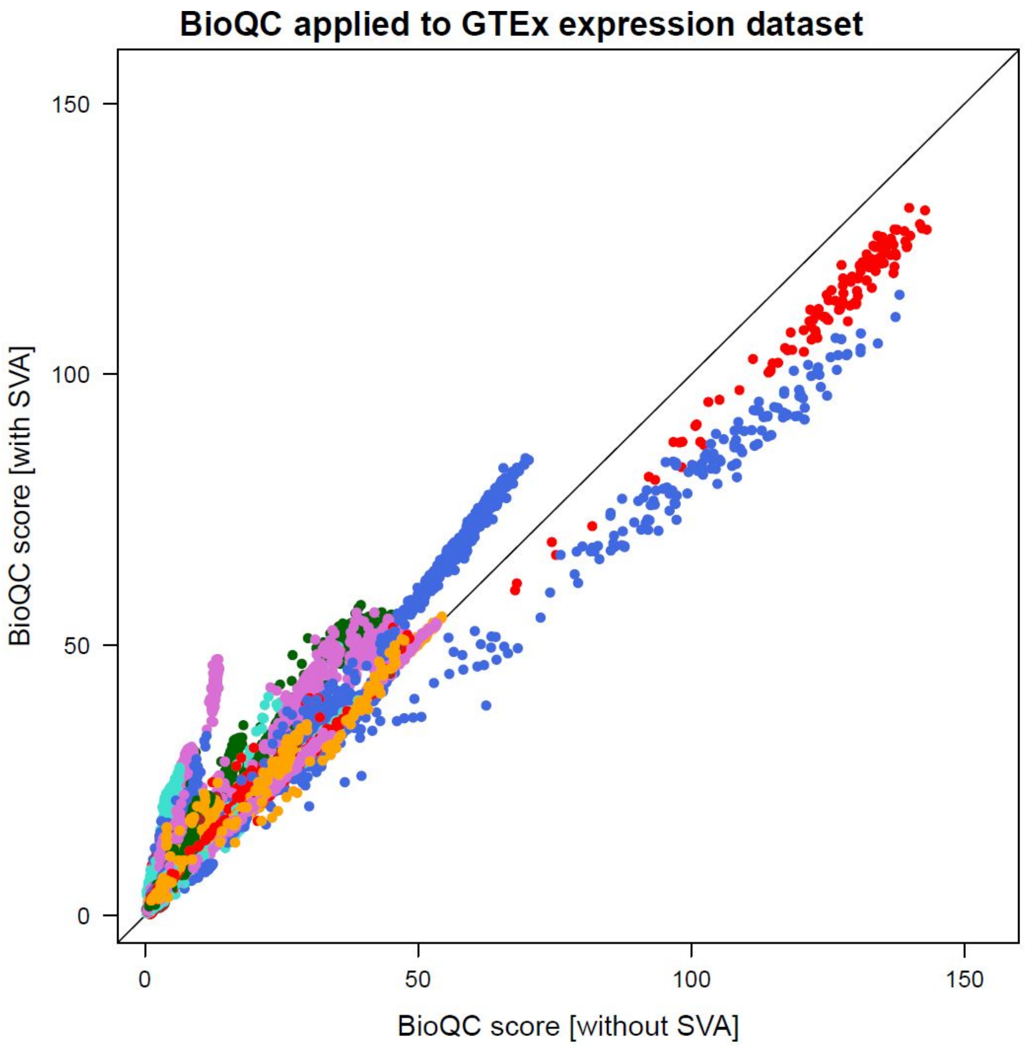

Figure S1: Scatterplot of *BioQC* s cores of original *B ioQC* signatures and sva-corrected signatures applied to the GTEx gene expression data (version 6, N=8555 samples). Each dot represents one tissue sample in GTEx, and the *x* and *y* coordinate represent the highest BioQC score of the original signature and of the sva-corrected signature, respectively. Distinct colours of dots represent different tissues reported by the GTEx database. We manually inspected the results and observe that for the majority of the cases the reported top-ranking tissue by *BioQC* is identical when using both signatures sets and is consistent with the tissue reported by the GTEx database.

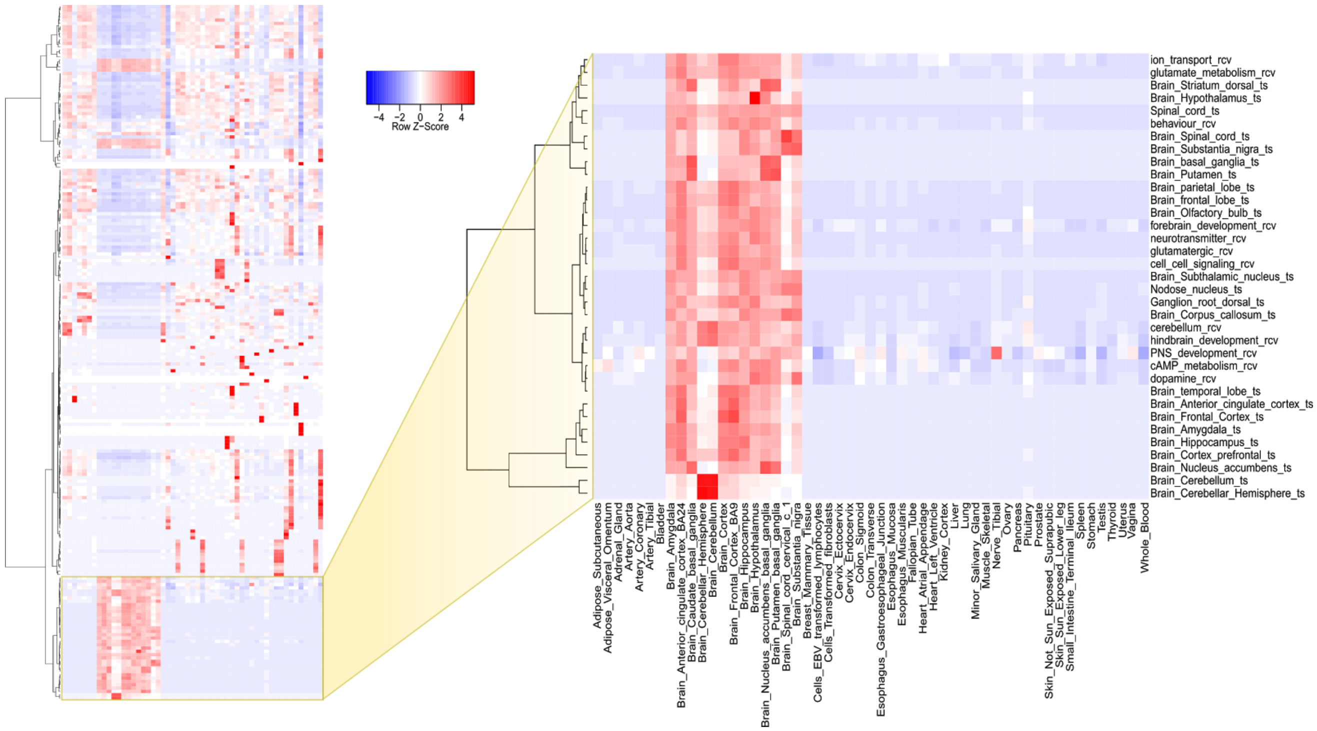

Figure S2: Tissue-enriched signatures and RCV signatures co-enrich in GTEx expression data. Left panel: heatmap showing the results of *BioQC* using both tissue signatures and RCV signatures that are based on biological processes defined by Gene Ontology (in rows) in representative tissue expression profiles derived from the GTEx database (in columns). Results are normalised to have zero mean and one standard deviation per row. Hierarchical clustering with Euclidean distance is performed on both rows and columns. Right panel: zoom-in of the co-clustering of tissue signatures associated with the nervous system and neuronal biological processes.
