# Supplementary material for: Detect tissue heterogeneity in gene expression data with BioQC
Source: BMC Genomics. 2017 Apr 4;18:277. doi: 10.1186/s12864-017-3661-2 (PMC5379536; doi:10.1186/s12864-017-3661-2)
Supplement: Supplementary file 2 — Supplementary Document 1. This document can also be assessed on the BioQC website under [27] respectively. (ZIP 98 kb) [file 12864_2017_3661_MOESM2_ESM.zip › SuppDoc-1.html]

BioQC Algorithm: Speeding up the Wilcoxon-Mann-Whitney Test | BioQC Documentation and Examples


Toggle navigation

  BioQC

- News
- @github
- Feedback


- BioQC
- Getting Started
  - Basic Usage
  - Gene Set Analysis
- The Algorithm
  - Advantages of the WMW-test
  - Speeding up the WMW-test
- Case Studies
  - Benchmark
  - Kidney Expression Example


# BioQC Algorithm: Speeding up the Wilcoxon-Mann-Whitney Test

- Algorithmic improvements
- Time benchmark
- Conclusion
- R Session Info

Supplementary Information for “Detect issue heterogenity in gene
expression data with *BioQC*” (Jitao
David Zhang, Klas Hatje, Gregor
Sturm, Clemens Broger, Martin Ebeling, Martine Burtin, Fabiola Terzi,
Silvia Ines Pomposiello and Laura Badi)

In this vignette, we explain the underlaying algorithmic details of our
implementation of the Wilcoxon-Mann-Whitney test. The source code used
to produce this document can be found in the github repository
BioQC.

*BioQC* is a R/Bioconductor package to detect tissue heterogeneity from
high-throughput gene expression profiling data. It implements an
efficient Wilcoxon-Mann-Whitney test, and offers tissue-specific gene
signatures that are ready to use ‘out of the box’.

## Algorithmic improvements

The Wilcoxon-Mann-Whitney (WMW) test is a non-parametric statistical
test to test if median values of two population are equal or not. Unlike
the t-test, it does not require the assumption of normal distributions,
which makes it more robust against noise.

We improved the computational efficiency of the Wilcoxon-Mann-Whitney
test in comparison to the native R implementation based on three
modifications:

1. The approximative WMW-statistic (Zar, J. H. (1999).
   Biostatistical analysis. Pearson Education India. *pp.* 174-177)
   is used. The differences to the exact version are negligible for
   high-throughput gene expression data.
2. The core algorithm is implemented in C instead of R as
   programming language.
3. BioQC avoids futile expensive sorting operations.

While (1) and (2) are straightforward, we elaborate (3) in the
following.

Let *W**a*, *b* be the approximative WMW test of two gene
vectors *a*, *b*, where *a* is the gene set of interest, typically
containing less than a few hundreds of genes, and *b* is the set of all
genes outside the gene set (*background genes*) typically containing
>10000 genes. In the context of BioQC, the gene sets are referred to
as *tissue signatures*.

Given an *m* × *n* input matrix of gene expression data with *m* genes
and *n* samples *s*1, …, *s**n*, and *k* gene sets
*d*1, …, *d**k*, the WMW-test needs to be applied
for each sample *s**i*, *i* ∈ 1..*n* and each gene set
*d**j*, *j* ∈ 1..*k*. The runtime of the WMW-test is
essentially determined by the sorting operation on the two input
vectors. Using native R `wilcox.test`, the vectors *a* and *b* are
sorted individually for each gene set. However, in the context of gene
set analysis, this is futile, as the (large) background set changes
insignificantly in relation to the (small) gene set, when testing
different gene sets on the same sample.

Therefore, we approximate the WMW-test by extending *b* to all genes in
the sample, keeping the background unchanged when testing multiple gene
sets. Like this, *b* has to be sorted only once per sample. The
individual gene sets still need to be sorted, which is not a major
issue, as they are small in comparison to the set of background genes.

**Figure 1**: BioQC speeds up the Wilcoxon-Mann-Whitney test by avoiding
futile sorting operations on the same sample.

## Time benchmark

To demonstrate BioQC’s superior performance, we apply both BioQC and the
native R `wilcox.test` to random expression matrices and measure the
runtime.

«««< HEAD
We setup random expression matrices of 20534 human protein-coding genes
=======
We setup random expression matrices of 20545 human protein-coding genes
»»»> 401feef2fb14a433e55d6520074f8fa12a66b943
of 1, 5, 10, 50, or 100 samples. Genes are *i*.*i*.*d* distributed
following 𝒩(0, 1). The native R and the *BioQC* implementations of the
Wilcoxon-Mann-Whitney test are applied to the matrices respectively.

The numeric results of both implementations, `bioqcNumRes` (from
*BioQC*) and `rNumRes` (from *R*), are equivalent, as shown by the next
command.

```
expect_equal(bioqcNumRes, rNumRes)
```

The *BioQC* implementation is more than 500 times faster: while it takes
about one second for BioQC to calculate enrichment scores of all 155
signatures in 100 samples, the native R implementation takes about 20
minutes:

**Figure 2**: Time benchmark results of BioQC and R implementation of
Wilcoxon-Mann-Whitney test. Left panel: elapsed time in seconds
(logarithmic Y-axis). Right panel: ratio of elapsed time by two
implementations. All results achieved by a single thread on in a RedHat
Linux server.

## Conclusion

We have shown that *BioQC* achieves identical results as the native
implementation in two orders of magnitude less time. This renders
*BioQC* a highly efficient tool for quality control of large-scale
high-throughput gene expression data.

## R Session Info

```
sessionInfo()
```

```
## R version 3.3.1 (2016-06-21) <<<<<<< HEAD
## Platform: x86_64-pc-linux-gnu (64-bit)
## Running under: Red Hat Enterprise Linux Server release 6.3 (Santiago) =======
## Platform: i686-pc-linux-gnu (32-bit)
## Running under: Linux Mint 18 >>>>>>> 401feef2fb14a433e55d6520074f8fa12a66b943
## 
## locale:
##  [1] LC_CTYPE=de_CH.UTF-8       LC_NUMERIC=C              
##  [3] LC_TIME=de_CH.UTF-8        LC_COLLATE=de_CH.UTF-8    
##  [5] LC_MONETARY=de_DE.UTF-8    LC_MESSAGES=de_CH.UTF-8   
##  [7] LC_PAPER=de_DE.UTF-8       LC_NAME=C                 
##  [9] LC_ADDRESS=C               LC_TELEPHONE=C            
## [11] LC_MEASUREMENT=de_DE.UTF-8 LC_IDENTIFICATION=C       
## 
## attached base packages:
## [1] stats4    parallel  methods   stats     graphics  grDevices utils    
## [8] datasets  base     
## 
## other attached packages:
##  [1] rbenchmark_1.0.0     gplots_3.0.1         gridExtra_2.2.1      <<<<<<< HEAD
##  [4] latticeExtra_0.6-28  RColorBrewer_1.1-2   lattice_0.20-33     
##  [7] hgu133plus2.db_3.2.3 org.Hs.eg.db_3.3.0   AnnotationDbi_1.34.4
## [10] IRanges_2.6.1        S4Vectors_0.10.2     BioQC_1.02.1        
## [13] Biobase_2.32.0       BiocGenerics_0.18.0  Rcpp_0.12.5         
## [16] testthat_1.0.2       knitr_1.13          
## 
## loaded via a namespace (and not attached):
##  [1] formatR_1.4        bitops_1.0-6       tools_3.3.1       
##  [4] digest_0.6.9       RSQLite_1.0.0      evaluate_0.9      
##  [7] gtable_0.2.0       DBI_0.4-1          yaml_2.1.13       
## [10] stringr_1.0.0      gtools_3.5.0       caTools_1.17.1    
## [13] grid_3.3.1         R6_2.1.2           rmarkdown_1.0     
## [16] gdata_2.17.0       magrittr_1.5       codetools_0.2-14  
## [19] htmltools_0.3.5    KernSmooth_2.23-15 stringi_1.1.1      =======
##  [4] latticeExtra_0.6-28  RColorBrewer_1.1-2   lattice_0.20-34     
##  [7] hgu133plus2.db_3.2.2 org.Hs.eg.db_3.2.3   RSQLite_1.0.0       
## [10] DBI_0.5-1            AnnotationDbi_1.32.3 IRanges_2.4.8       
## [13] S4Vectors_0.8.11     BioQC_1.02.1         Biobase_2.30.0      
## [16] BiocGenerics_0.16.1  Rcpp_0.12.8          testthat_1.0.2      
## [19] knitr_1.15          
## 
## loaded via a namespace (and not attached):
##  [1] codetools_0.2-15   digest_0.6.10      htmltools_0.3.5   
##  [4] R6_2.2.0           assertthat_0.1     grid_3.3.1        
##  [7] bitops_1.0-6       stringr_1.1.0      gdata_2.17.0      
## [10] highr_0.6          tibble_1.2         KernSmooth_2.23-15
## [13] stringi_1.1.2      magrittr_1.5       caTools_1.17.1    
## [16] rmarkdown_1.1      evaluate_0.10      gtable_0.2.0      
## [19] yaml_2.1.13        tools_3.3.1        gtools_3.5.0       >>>>>>> 401feef2fb14a433e55d6520074f8fa12a66b943
## [22] crayon_1.3.2
```

---


©2016 F. Hoffmann-La Roche AG. All rights reserved.   
Site last generated: Nov 23, 2016
