# Supplementary material for: Detect tissue heterogeneity in gene expression data with BioQC
Source: BMC Genomics. 2017 Apr 4;18:277. doi: 10.1186/s12864-017-3661-2 (PMC5379536; doi:10.1186/s12864-017-3661-2)
Supplement: Supplementary file 3 — Supplementary Document 2. This document can also be assessed on the BioQC website under [28] respectively. (ZIP 305 kb) [file 12864_2017_3661_MOESM3_ESM.zip › SuppDoc-2.html]

BioQC-benchmark: Testing Efficiency, Sensitivity and Specificity of BioQC on simulated and real-world data | BioQC Documentation and Examples


Toggle navigation

  BioQC

- News
- @github
- Feedback


- BioQC
- Getting Started
  - Basic Usage
  - Gene Set Analysis
- The Algorithm
  - Advantages of the WMW-test
  - Speeding up the WMW-test
- Case Studies
  - Benchmark
  - Kidney Expression Example


# BioQC-benchmark: Testing Efficiency, Sensitivity and Specificity of BioQC on simulated and real-world data

- Experiment setup
- Sensitivity Benchmark
- Mixing Benchmark
  - Dataset selection and quality control
  - An example of weighted mixing: heart and jejunum
  - Pairwise Mixing
  - Comparing BioQC with Principal Component Analysis (PCA)
- R Session Info

Supplementary Information for “Detect issue heterogenity in gene
expression data with *BioQC*” (Jitao
David Zhang, Klas Hatje, Clemens
Broger, Martin Ebeling, Martine Burtin, Fabiola Terzi, Silvia Ines
Pomposiello, Gregor Sturm and Laura Badi)

In this vignette, we perform simulations with both model-generated and
real-world data using *BioQC*. We show that *BioQC* is a sensitive
method to detect tissue heterogeneity from high-throughput gene
expression data. The source code used to produce this document can be
found in the github repository
BioQC-example.

*BioQC* is a R/Bioconductor package to detect tissue heterogeneity from
high-throughput gene expression profiling data. It implements an
efficient Wilcoxon-Mann-Whitney test, and offers tissue-specific gene
signatures that are ready to use ‘out of the box’.

## Experiment setup

In this document, we perform two simulation studies with *BioQC*:

- **Sensitivity benchmark** tests the sensitivity and specificity of
  *BioQC* detecting tissue heterogeneity using model-generated,
  simulated data;
- **Mixing benchmark** tests the sensitivity and specificity of
  *BioQC* using simulated contamination with real-world data.

All source code that is needed to reproduce the results can be found in
the `.Rmd` file generating this document.

```
library(BioQC)
library(hgu133plus2.db) ## to simulate an microarray expression dataset
library(lattice)
library(latticeExtra)
library(gridExtra)
library(GEOquery)
library(xtable)
library(gplots)

pdf.options(family="ArialMT", useDingbats=FALSE)

set.seed(1887)

## list human genes
humanGenes <- unique(na.omit(unlist(as.list(hgu133plus2SYMBOL))))

## read tissue-specific gene signatures
gmtFile <- system.file("extdata/exp.tissuemark.affy.roche.symbols.gmt",
                       package="BioQC")
gmt <- readGmt(gmtFile)
```

## Sensitivity Benchmark

We next asked the question how sensitive *BioQC* is to expression
changes of tissue signature genes. Similar to the previous simulation,
we create random expression matrices. While keeping all other genes
*i*.*i*.*d* normally distributed following 𝒩(0, 1), we dedicatedly
increase the expression of genes in one randomly selected tissue
signature (ovary, with 43 genes) by different amplitudes: these genes’
expression levels are randomly drawn from different normal distributions
with varying expectation and constant variance between 𝒩(0, 1) and
𝒩(3, 1). To test the robustness of the algorithm, 10 samples are
generated for each mean expression difference value.

Sensitivity benchmark. Expression levels of genes in the ovary signature
are dedicately sampled randomly from normal distributions with different
mean values. Left panel: enrichment scores reported by *BioQC* for the
ovary signature, plotted against the differences in mean expression
values; Right panel: rank of ovary enrichment scores in all 155
signatures plotted against the difference in mean expression values.

The above figure visualizes the distribution of enrichment scores and
their ranks dependent on the mean expression difference between ovary
signature genes and background genes. As soon as the expression of
signature genes increases by a very moderate ampltiude 1*σ*, *BioQC*
will identify the gene set as the highest-ranking signature. A even
stronger difference in expression will lead to higher enrichment scores
but no change in the rank.

The results suggest that *BioQC* is sensitive even to moderate changes
in the average expression of a gene set.

## Mixing Benchmark

The sensitivity benchmark above suffers from the limitation that the
distributions of gene expression are not physiological. To overcome
this, we designed and performed a benchmark by *in silico* mixing
expression profiles with weighted linear combination, thereby mimicking
tissue contamination.

Given the expression profile of a sample of tissue A
(**Y***A*), and that of a sample of tissue B
(**Y***B*), the weighted linear mixing produces a new profile
**Y** = *ω***Y****A** + (1 − *ω*)**Y****B**, where
*ω* ∈ [0, 1]. In essence the profiles of two tissue types are linearly
mixed in different proportions, which simulates varying severities of
contaminations. We asked whether BioQC could detect such mixings, and if
so, how sensitive is the method.

### Dataset selection and quality control

In order to avoid over-fitting of signatures derived from human
expression data, we decided to use a normal tissue expression dataset
from a non-human mammal species, because it has been shown that
tissue-preferential expression patterns tend to be conserved between
mammal species. We identified a dataset of *Canis lupus familiaris*
(dog), which is publicly available in Gene Expression Omnibus
(GDS4164).

In this study, the authors examined 39 samples from 10 pathologically
normal tissues (liver, kidney, heart, lung, brain, lymph node, spleen,
jejunum, pancreas, and skeletal muscle) of four dogs (with one pancreas
sample missing). We downloaded the data, and performed minimal
pre-processing: for multiple probesets that map to same genes, we kept
the one with the highest average expression level and removed the rest.
The resulting dataset contained expression of 16797 genes. BioQC was
applied to the dataset to test whether there are major contamination
issues. The results, including tissues reported by the authors, and the
BioQC tissue signatures with the highest and second-highest scores, are
reported in the following table:

|  | Label | BioQC.best | BioQC.second |
| --- | --- | --- | --- |
| GSM502573 | Brain | Spinal\_cord | Nodose\_nucleus |
| GSM502574 | Brain | Brain\_Cortex\_prefrontal | Brain\_Amygdala |
| GSM502575 | Brain | Brain\_Cortex\_prefrontal | Brain\_Amygdala |
| GSM502576 | Brain | Brain\_Cortex\_prefrontal | Brain\_Amygdala |
| GSM502577 | Heart | Muscle\_cardiac | Muscle\_skeletal |
| GSM502578 | Heart | Muscle\_cardiac | Muscle\_skeletal |
| GSM502579 | Heart | Muscle\_cardiac | Muscle\_skeletal |
| GSM502580 | Heart | Muscle\_cardiac | Muscle\_skeletal |
| GSM502581 | Jejunum | Intestine\_small | Intestine\_Colon |
| GSM502582 | Jejunum | Intestine\_small | Intestine\_Colon |
| GSM502583 | Jejunum | Intestine\_small | Intestine\_Colon |
| GSM502584 | Jejunum | Intestine\_small | Intestine\_Colon |
| GSM502585 | Kidney | Kidney | Kidney\_Renal\_Cortex |
| GSM502586 | Kidney | Kidney | Kidney\_Renal\_Cortex |
| GSM502587 | Kidney | Kidney | Kidney\_Renal\_Cortex |
| GSM502588 | Kidney | Kidney | Kidney\_Renal\_Cortex |
| GSM502589 | Liver | Liver | Liver |
| GSM502590 | Liver | Liver | Liver |
| GSM502591 | Liver | Liver | Liver |
| GSM502592 | Liver | Liver | Liver |
| GSM502593 | Lung | Lung | Monocytes |
| GSM502594 | Lung | Monocytes | Lung |
| GSM502595 | Lung | Lung | Monocytes |
| GSM502596 | Lung | Monocytes | Lung |
| GSM502597 | LymphNode | Lymphocyte\_B\_FOLL | Lymphocytes\_T\_H |
| GSM502598 | LymphNode | Lymphocyte\_B\_FOLL | Lymphocytes\_T\_H |
| GSM502599 | LymphNode | Lymphocyte\_B\_FOLL | Lymphocytes\_T\_H |
| GSM502600 | LymphNode | Lymphocyte\_B\_FOLL | Lymphocytes\_T\_H |
| GSM502601 | Pancreas | Pancreas\_Islets | Pancreas |
| GSM502602 | Pancreas | Pancreas\_Islets | Pancreas |
| GSM502603 | Pancreas | Pancreas\_Islets | Pancreas |
| GSM502604 | SkeletalMuscle | Muscle\_skeletal | Muscle\_cardiac |
| GSM502605 | SkeletalMuscle | Muscle\_skeletal | Muscle\_cardiac |
| GSM502606 | SkeletalMuscle | Muscle\_skeletal | Muscle\_cardiac |
| GSM502607 | SkeletalMuscle | Muscle\_skeletal | Muscle\_cardiac |
| GSM502608 | Spleen | Monocytes | Lymphocyte\_B\_FOLL |
| GSM502609 | Spleen | Monocytes | Lymphocyte\_B\_FOLL |
| GSM502610 | Spleen | Monocytes | Erythroid\_cells |
| GSM502611 | Spleen | Monocytes | Myeloblast |

By comparing the tissue labels provided by the authors and the
predictions of *BioQC*, we notice that in most cases the two match well
(despite of ontological differences). In three cases (sample ID
GSM502573, GSM502594, and GSM502596) though, there seem to be intriguing
differences, which might be explained by different sampling procedures
or immune cell infiltration. We will however in this vignette not
further explore them. These three samples are removed from the
simulation procedures.

### An example of weighted mixing: heart and jejunum

As an example, we take average expression of heart and jejunum samples,
and mix them by different compositions. This allows us comparing
enrichment scores and their ranks when the expression profiles of heart
and jejunum are mixed *in silico*:

Results of a mixing case study. Left panel: *BioQC* enrichment scores of
small intestine and cardiac muscle varying upon different proportions of
jejunum; Right panel: ranks of enrichment scores varying upon different
proportions of jejunum.

We observe that with as little as 5% contamination of heart tissue in
jejunum samples (rightmost in the right panel), the rank of heart
signature jumps from 34 to 9; 10% and 20% contamination will further
enhance the rank to 4 and 3 respectively. If we start from the other
end, namely assuming jejunum contamination in heart samples, the BioQC
algorithms ranks jejunum the 7th only when there are more than 25%
contamination. If we set enrichment score equal or over 3 as the
threshold of calling a suspected contamination event (*p* < 0.001 in
the one-sided Wilcoxon-Mann-Whitney test), it takes about 10% heart in
jejunum tissue or about 30% jejunum tissue in heart to make a call. It
means the sensitivity of contamination detection is not symmetric
between tissues: contamination by tissues with distinct expression
patterns (such as heart) are easier to be detected than contamination by
tissues with less distinct expression patterns (such as small
intestine).

While it is difficult to quantify the absolute sensitivity of
contamination detection, it is apparent that if the enrichment score of
a unforeseen tissue is very high (or ranked high), one may suspect
potential contamination. Also, if there are replicates of samples from
the same tissue, a higher value in one sample compared with the other
samples suggests a contamination or infiltration incident.

### Pairwise Mixing

Following the heart-jejunum example, we performed all 45 pairwise mixing
experiments, producing weighted linear combinations of gene expression
profiles of each pair of tissues (excluding self-mixing). The results
are summaried in a heatmap:

Results of the pairwise mixing experiment. Each cell represents the
minimal percentage of tissue of the column as contamination in the
tissue of the row that can be detected by *BioQC*. No values are
available for cells on the diagonal because self-mixing was excluded.
Heart and skeletal muscle are very close to each other and therefore
their detection limit is not considered.

The heatmap visualization summarizes the detection limit of
contamination of each pair of tissues. Take the cell in row 1 column 2
from top left: its value (0.15) means that if there are 15% or more
contamination by heart in the brain sample, *BioQC* will be able to
detect it (with the threshold enrichment score ≥3 or the rank ≤10),
because the enrichment score is equal to or larger than 3, or the heart
tissue signature ranks in the top 10 of all tissue signatures.

Take another cell in row 2 column 1 from top left: its value (0.5) means
that if there are 50% or more contanmination by brain in a heart sample,
*BioQC* will be able to detect it. Here we observe the asymmetry again
that we observed before with the heart/jejenum example: while it is
realtively easy to identify heart contamination of a brain sample, it is
more difficult to identify brain contamination of a heart sample in this
dataset.

The average detection limits of tissues as contamination sources are
listed in the following table. The values are derived from median values
of each column in the heatmap, except for diagonal and missing elements.

| Tissue | MedianDetectionLimit |
| --- | --- |
| Brain | 52.22% |
| Heart | 10.62% |
| Jejunum | 19.44% |
| Kidney | 23.75% |
| Liver | 11.87% |
| Lung | 31.67% |
| LymphNode | 30.63% |
| Pancreas | 17.22% |
| SkeletalMuscle | 15.00% |
| Spleen | 13.57% |

Interestingly, brain samples are a special case: if they contaminate
other tissues, it is more difficult to identify (but not other way
around). It can be partially explained by the experiment design: Briggs
*et al.* profiled the whole cerebrum, whereas in *BioQC* there are 22
distinct gene sets assigned to distinct brain regions. Though the
prefrontal cortex signature scored highest in the canine brain samples,
its score is relative low (7.45), and the genes in the signature are not
too far away from the background genes:

Tissue-specific genes’ expression in respective average tissue profiles.
For each tissue (*e.g.* brain), we calculate the median ratio of gene
expression level of specific genes over the median expression level of
background genes. The value reflects the specificity of tissue-specific
genes in respective tissues. Likely due to the sampling of different
brain regions, the score of brain ranks the lowest.

Therefore only a strong contamination by brain in this dataset will be
detected by the given threshold. We expect that if prefrontal cortex
instead of cerebrum sample was profiled, the mixing profile of brain
will be similar to other organs. This needs to be tested in other
datasets.

Apart from that, most *in silico* contamination events are detectable in
this dataset, with median detection limit around 0.2. This suggests that
*BioQC* is sensitive towards tissue heterogeneity in physiological
settings.

# Conclusions

Benchmark studies with similated and real-world data demonstrate that
*BioQC* is an efficient and sensitive method to detect tissue
heterogeneity from high-throughput gene expression data.

# Appendix

### Comparing BioQC with Principal Component Analysis (PCA)

In the context of the dog transcriptome dataset, we can compare the
results of principal component analysis (PCA) with that of *BioQC*:

Sample separation revealed by principal component analysis (PCA) of the
dog transcriptome dataset.

PCA sugggests that samples from each tissue tend to cluster together, in
line with the *BioQC* results. In addition, PCA reveals that tissues
with cells of similar origins cluster together, such as skeletal muscle
and heart. As expected, one brain sample and two lung samples seem to be
different from other samples of the same cluster, which are consistent
with the *BioQC* findings; however, unlike BioQC, PCA does not provide
information on what are potential contamination/infiltration casues.

Therefore, we believe *BioQC* complements existing unsupervised methods
to inspect quality of gene expression data.

## R Session Info

```
sessionInfo()
```

```
## R version 3.3.1 (2016-06-21)
## Platform: i686-pc-linux-gnu (32-bit)
## Running under: Linux Mint 18
## 
## locale:
##  [1] LC_CTYPE=de_CH.UTF-8       LC_NUMERIC=C              
##  [3] LC_TIME=de_CH.UTF-8        LC_COLLATE=de_CH.UTF-8    
##  [5] LC_MONETARY=de_DE.UTF-8    LC_MESSAGES=de_CH.UTF-8   
##  [7] LC_PAPER=de_DE.UTF-8       LC_NAME=C                 
##  [9] LC_ADDRESS=C               LC_TELEPHONE=C            
## [11] LC_MEASUREMENT=de_DE.UTF-8 LC_IDENTIFICATION=C       
## 
## attached base packages:
## [1] stats4    parallel  methods   stats     graphics  grDevices utils    
## [8] datasets  base     
## 
## other attached packages:
##  [1] gplots_3.0.1         xtable_1.8-2         GEOquery_2.36.0     
##  [4] gridExtra_2.2.1      latticeExtra_0.6-28  RColorBrewer_1.1-2  
##  [7] lattice_0.20-34      hgu133plus2.db_3.2.2 org.Hs.eg.db_3.2.3  
## [10] RSQLite_1.0.0        DBI_0.5-1            AnnotationDbi_1.32.3
## [13] IRanges_2.4.8        S4Vectors_0.8.11     BioQC_1.02.1        
## [16] Biobase_2.30.0       BiocGenerics_0.16.1  Rcpp_0.12.8         
## [19] knitr_1.15          
## 
## loaded via a namespace (and not attached):
##  [1] magrittr_1.5       highr_0.6          stringr_1.1.0     
##  [4] caTools_1.17.1     tools_3.3.1        grid_3.3.1        
##  [7] gtable_0.2.0       KernSmooth_2.23-15 gtools_3.5.0      
## [10] htmltools_0.3.5    yaml_2.1.13        assertthat_0.1    
## [13] digest_0.6.10      tibble_1.2         bitops_1.0-6      
## [16] RCurl_1.95-4.8     evaluate_0.10      rmarkdown_1.1     
## [19] gdata_2.17.0       stringi_1.1.2      XML_3.98-1.5
```

---


©2016 F. Hoffmann-La Roche AG. All rights reserved.   
Site last generated: Nov 22, 2016
