# Supplementary material for: Detect tissue heterogeneity in gene expression data with BioQC
Source: BMC Genomics. 2017 Apr 4;18:277. doi: 10.1186/s12864-017-3661-2 (PMC5379536; doi:10.1186/s12864-017-3661-2)
Supplement: Supplementary file 4 — Supplementary Document 3. This document can also be assessed on the BioQC website under [29] respectively. (ZIP 325 kb) [file 12864_2017_3661_MOESM4_ESM.zip › SuppDoc-3.html]

BioQC-kidney: The kidney expression example | BioQC Documentation and Examples


Toggle navigation

  BioQC

- News
- @github
- Feedback


- BioQC
- Getting Started
  - Basic Usage
  - Gene Set Analysis
- The Algorithm
  - Advantages of the WMW-test
  - Speeding up the WMW-test
- Case Studies
  - Benchmark
  - Kidney Expression Example


# BioQC-kidney: The kidney expression example

- Introduction
- Importing the data
- Running BioQC
- Validation with quantitative RT-PCR
- Impact of sample removal on differential gene expression analysis
- R Session Info

Supplementary Information for “Detect issue heterogenity in gene
expression data with *BioQC*” (Jitao
David Zhang, Klas Hatje, Clemens
Broger, Martin Ebeling, Martine Burtin, Fabiola Terzi, Silvia Ines
Pomposiello, Gregor Sturm and Laura Badi)

## Introduction

In this vignette, we demonstrate the use of *BioQC* with a case study
where mouse kidney samples were profiled for gene expression. Results of
BioQC pointed to potential tissue heterogeneity caused by pancreas
contamination which was confirmed by qRT-PCR experiments. Source code
and data needed to reproduce this document can be found in the github
repository BioQC-example.

This is a supplementary documentation of *BioQC*, a R/Bioconductor
package used to detect tissue heterogeneity from high-throughput gene
expression profiling data with tissue-specific gene signatures. For its
basic use please refer to the documentation and vignettes shipped along
with the package, or to the other vignette
bioqc-simulation which applies the algorithm to
simulated datsets. Here we demonstrate its use with a real biological
data set, which is not included in the package distribution due to size
limitations.

## Importing the data

First, we load the package, the tissue-specific gene signatures, and the
expression data into the R session.

```
library(BioQC)

gmtFile <- system.file("extdata/exp.tissuemark.affy.roche.symbols.gmt",
                        package="BioQC")
gmt <- readGmt(gmtFile)
```

```
file <- "data/bioqc-nephrectomy.RData"
load(file)
print(eset)
```

```
## ExpressionSet (storageMode: lockedEnvironment)
## assayData: 34719 features, 25 samples 
##   element names: exprs 
## protocolData: none
## phenoData
##   sampleNames: FSP5.FVB.NJ.Sham.Placebo FS2.FVB.NJ.Sham.Control
##     ... FN6.FVB.NJ.Nephrectomy.Control (25 total)
##   varLabels: Experiment.name INDIVIDUALNAME ... Elastase (7 total)
##   varMetadata: labelDescription
## featureData
##   featureNames: 1415670_at 1415671_at ...
##     AFFX-TransRecMur/X57349_M_at (34719 total)
##   fvarLabels: GeneID GeneSymbol OrigGeneID OrigGeneSymbol
##   fvarMetadata: labelDescription
## experimentData: use 'experimentData(object)'
## Annotation:
```

The dataset contains expression of 34719 genes in 25 samples. The
expression profile was normalized with RMA normalization. The signals
were also `log2`-transformed; however, this step does not affect the
result of *BioQC* since it is essentially a non-parametric statistical
test.

## Running BioQC

Next we run the core function of the *BioQC* package, `wmwTest`, to
perform the analysis.

```
system.time(bioqcRes <- wmwTest(eset, gmt,
                                 valType="p.greater"))
```

```
##    user  system elapsed 
##   1.664   1.088   3.431
```

The function returns *one-sided* *p*-values of Wilcoxon-Mann-Whitney
test. We next visualize this metric after transformation.

```
bioqcResFil <- filterPmat(bioqcRes, 1E-8)
bioqcAbsLogRes <- absLog10p(bioqcResFil)
```

By closer examination we find that the expression of pancreas and
adipose specific genes is significantly enriched in samples 23-25:

```
library(RColorBrewer)
heatmap(bioqcAbsLogRes, Colv=NA, Rowv=TRUE,
        cexRow=0.85,
        col=rev(brewer.pal(7, "RdBu")),
        labCol=1:ncol(bioqcAbsLogRes))
```

BioQC scores (defined as abs(log10(p))) of the samples visualized in
heatmap. Red and blue indicate high and low scores respectively.

Visual inspection reveals that there might be contaminations in samples
23-25, potentially by pancreas and adipose tissue.

```
filRes <- bioqcAbsLogRes[c("Kidney_NGS_RNASEQATLAS_0.6_3",
                           "Pancreas_Islets_NR_0.7_3"),]
matplot(t(filRes), pch=c("K", "P"), type="b", lty=1L,
        ylab="BioQC score", xlab="Sample index")
```

BioQC scores (defined as abs(log10(p))) of the samples. K and P
represent kidney and pancreas signature scores respectively.

## Validation with quantitative RT-PCR

To confirm the hypothesis generated by *BioQC*, we performed qRT-PCR
experiments to test two pancreas-specific genes’ expression in the same
set of samples. Note that the two genes (amylase and elastase) are not
included in the signature set provided by BioQC.

The results are shown in the figure below. It seems likely that sample
23-25 are contaminated by nearby pancreas tissues when the kidney was
dissected. Potential contamination by adipose tissues remains to be
tested.

```
amylase <- eset$Amylase
elastase <- eset$Elastase
pancreasScore <- bioqcAbsLogRes["Pancreas_Islets_NR_0.7_3",]
par(mfrow=c(1,2), mar=c(3,3,1,1), mgp=c(2,1,0))
plot(amylase~pancreasScore, log="y", pch=21, bg="red",
     xlab="BioQC pancreas score", ylab="Amylase")
text(pancreasScore[23:25],amylase[23:25], 23:25, pos=1)
plot(elastase~pancreasScore, log="y", pch=21, bg="red",
     xlab="BioQC pancreas score", ylab="Elastase")
text(pancreasScore[23:25],elastase[23:25], 23:25, pos=1)
```

Correlation between qRT-PCR results and BioQC pancreas score

## Impact of sample removal on differential gene expression analysis

In this study, four mice of the *FVB/NJ* strain received nephrectomy
operation and treatment of Losartan, an angiotensin II receptor
antagonist drug, and four mice reveiced an sham operation and Losartan.
Within the Nephrectomy+Losartan group, one sample (index 24) is possibly
contaminated by pancreas. Suppose now we are interested in the
differential gene expression between the conditions. We now run the
analysis twice, once with and once without the contaminated sample, to
study the impact of removing heterogenous samples detected by *BioQC*.

```
library(limma)
```

```
isNeph <- with(pData(eset), Strain=="FVB/NJ" &
                 TREATMENTNAME %in% c("Nephrectomy-Losartan", "Sham-Losartan"))
isContam <- with(pData(eset), INDIVIDUALNAME %in% c("BN7", "FNL8", "FN6"))
esetNephContam <- eset[,isNeph]
esetNephExclContam <- eset[, isNeph & !isContam]
getDEG <- function(eset) {
  group <- factor(eset$TREATMENTNAME, levels=c("Sham-Losartan","Nephrectomy-Losartan"))
  design <- model.matrix(~group)
  colnames(design) <- c("ShamLo", "NephLo")
  contrast <- makeContrasts(contrasts="NephLo", levels=design)
  exprs(eset) <- normalizeBetweenArrays(log2(exprs(eset)))
  fit <- lmFit(eset, design=design)
  fit <- contrasts.fit(fit, contrast)
  fit <- eBayes(fit)
  tt <- topTable(fit, n=nrow(eset))
  return(tt)
}

esetNephContam.topTable <- getDEG(esetNephContam)
esetNephExclContam.topTable <- getDEG(esetNephExclContam)
esetFeats <- featureNames(eset)
esetNephTbl <- data.frame(feature=esetFeats,
                          OrigGeneSymbol=esetNephContam.topTable[esetFeats,]$OrigGeneSymbol,
                          GeneSymbol=esetNephContam.topTable[esetFeats,]$GeneSymbol,
                          Contam.logFC=esetNephContam.topTable[esetFeats,]$logFC,
                          ExclContam.logFC=esetNephExclContam.topTable[esetFeats,]$logFC)
par(mfrow=c(1,1), mar=c(3,3,1,1)+0.5, mgp=c(2,1,0))
with(esetNephTbl, smoothScatter(Contam.logFC~ExclContam.logFC,
                                xlab="Excluding one contaminating sample [logFC]",
                                ylab="Including one contaminating sample [logFC]"))
abline(0,1)
isDiff <- with(esetNephTbl, abs(Contam.logFC-ExclContam.logFC)>=2)
with(esetNephTbl, points(Contam.logFC[isDiff]~ExclContam.logFC[isDiff], pch=16, col="red"))
```

Log2 fold change (logFC) values reported by *limma* with one
contaminated sample included (y-axis) or excluded (x-axis). Genes
strongly affected by the contamination are indicated by red dots.

```
diffTable <- esetNephTbl[isDiff,]
diffGenes <- unique(diffTable[,"GeneSymbol"])
pancreasSignature <- gmt[["Pancreas_Islets_NR_0.7_3"]]$genes
diffGenesPancreas <- diffGenes %in% pancreasSignature
diffTable$isPancreasSignature <- diffTable$GeneSymbol %in% pancreasSignature
colnames(diffTable) <- c("Probeset", "GeneSymbol", "Human ortholog",
                         "Log2FC", "Log2FC (excl. contam.)",
                         "IsPancreasSignature")
diffTable <- diffTable[order(diffTable$Log2FC, decreasing=TRUE),]
```

We found that 22 probesets representing 17 genes are associated with
much stronger expression changes if the contaminated sample is not
excluded (table below). Not surprisingly almost all of these genes are
highly expressed in normal human pancreas tissues, and 13 genes belong
to the pancreas signature used by *BioQC*.

In summary, we observe that tissue heterogeneity can impact down-stream
analysis results and negatively affect reproducibility of gene
expression data if it remains overlooked. It underlines again the value
of applying *BioQC* as a first-line quality control tool.

## R Session Info

```
sessionInfo()
```

```
## R version 3.3.1 (2016-06-21)
## Platform: i686-pc-linux-gnu (32-bit)
## Running under: Linux Mint 18
## 
## locale:
##  [1] LC_CTYPE=de_CH.UTF-8       LC_NUMERIC=C              
##  [3] LC_TIME=de_CH.UTF-8        LC_COLLATE=de_CH.UTF-8    
##  [5] LC_MONETARY=de_DE.UTF-8    LC_MESSAGES=de_CH.UTF-8   
##  [7] LC_PAPER=de_DE.UTF-8       LC_NAME=C                 
##  [9] LC_ADDRESS=C               LC_TELEPHONE=C            
## [11] LC_MEASUREMENT=de_DE.UTF-8 LC_IDENTIFICATION=C       
## 
## attached base packages:
## [1] parallel  methods   stats     graphics  grDevices utils     datasets 
## [8] base     
## 
## other attached packages:
## [1] limma_3.26.9        RColorBrewer_1.1-2  BioQC_1.02.1       
## [4] Biobase_2.30.0      BiocGenerics_0.16.1 Rcpp_0.12.8        
## [7] knitr_1.15         
## 
## loaded via a namespace (and not attached):
##  [1] digest_0.6.10      assertthat_0.1     magrittr_1.5      
##  [4] evaluate_0.10      KernSmooth_2.23-15 highr_0.6         
##  [7] stringi_1.1.2      rmarkdown_1.1      tools_3.3.1       
## [10] stringr_1.1.0      yaml_2.1.13        htmltools_0.3.5   
## [13] tibble_1.2
```

---


©2016 F. Hoffmann-La Roche AG. All rights reserved.   
Site last generated: Nov 22, 2016
